# Generosity of Powers & Weightman

**Published:** 1880-03-20

**Authors:** 


### *GENEROSITY OF POWERS & WEIGHTMAN.*

This splendid and widely-known House, of Philadelphia, recently presented to the Museum of the Southern Medical College a beautiful walnut case, elegantly fitted up and filled with numerous samples of their chemicals. The chemicals are most beautiful, and unsurpassed for purity and perfection of manufacture. The token which they thus furnish of their sympathy and interest in this new College enterprise is but another of the many instances of the liberality of this great establishment, and we assure them it is warmly appreciated by the Faculty of the Institution and by our citizens generally.

